# Using machine learning and an ensemble of methods to predict kidney transplant survival

**DOI:** 10.1371/journal.pone.0209068

**Published:** 2019-01-09

**Authors:** Ethan Mark, David Goldsman, Brian Gurbaxani, Pinar Keskinocak, Joel Sokol

**Affiliations:** 1 H. Milton Stewart School of Industrial and Systems Engineering, Georgia Institute of Technology, Atlanta, Georgia, United States of America; 2 Centers for Disease Control and Prevention, Atlanta, Georgia, United States of America; INSERM, FRANCE

## Abstract

We used an ensemble of statistical methods to build a model that predicts kidney transplant survival and identifies important predictive variables. The proposed model achieved better performance, measured by Harrell’s concordance index, than the Estimated Post Transplant Survival model used in the kidney allocation system in the U.S., and other models published recently in the literature. The model has a five-year concordance index of 0.724 (in comparison, the concordance index is 0.697 for the Estimated Post Transplant Survival model, the state of the art currently in use). It combines predictions from random survival forests with a Cox proportional hazards model. The rankings of importance for the model’s variables differ by transplant recipient age. Better survival predictions could eventually lead to more efficient allocation of kidneys and improve patient outcomes.

## Introduction

In 2013, the Organ Procurement and Transplantation Network (OPTN) adopted a new kidney allocation system using the Estimated Post Transplant Survival (EPTS) score [1, 2]. Other kidney transplant survival models such as the Recipient Risk Score (RSS) [3] and Life Years from Transplant (LYFT) [4], have also been proposed by researchers. These techniques use a Cox proportional hazards model, which estimates the probability of a recipient’s post-transplant survival over a given time horizon [5]. The Cox proportional hazards model is the most widely used model for kidney transplant survival estimation [6]. Additional models include a Bayesian Belief Network (BBN) that was used to predict kidney graft failure [7].

We took a different approach, and used an ensemble of methods including random survival forests constructed from conditional inference trees. Our approach first clusters the data (e.g., into cohorts) and then chooses a model that achieves the best performance for each cluster. The advantage of combining different models to predict kidney transplant survival is that different models may work better than others on different cohorts of the data. We assessed the predictive accuracy of our proposed model using various metrics, including Harrell’s concordance index (C-index) [8], which is the percentage of patient pairs correctly “ranked” by the model based on their post-transplant survival duration in a given timeframe. The C-index for the proposed model is better than that of the EPTS model and other kidney transplant survival models proposed recently in the literature [2, 4, 6]. The results of the model applied to kidney transplant data are presented here, but the approach can be applied to other organs as well.

## Data

The dataset was provided by the United Network for Organ Sharing (UNOS) and consists of recipients who underwent kidney transplant surgery in the U.S. from 1987 to 2014 [9, 10]. The data includes both living and deceased donors, pediatric and adult recipients, and censored observations. An observation is censored when it does not record a transplant recipient’s survival duration after surgery; in these censored observations, the date of the last follow-up is recorded. All data in this study were fully anonymized prior to access by any of the authors. More information on the UNOS data and instructions for researchers to request this data can be found at https://unos.org/data/.

### Data preparation

**Fig 1:**
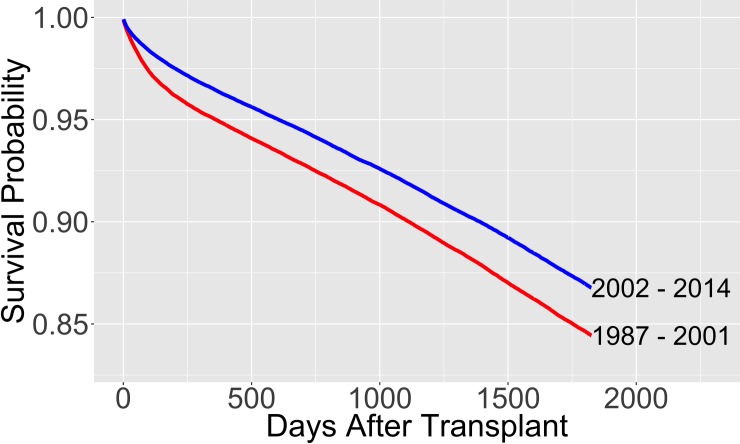
Survival probabilities of different transplant cohorts. The survival probabilities are calculated from the Kaplan-Meier estimate.

In 2003, the UNOS board of directors instructed the Kidney Allocation Review Subcommittee to review the kidney allocation system [1]. Hence, we tested the following hypothesis: There is a statistically significant difference between the survival curves of recipients who underwent a kidney transplant before and after 2002. A log-rank test and visual inspection of the survival curves verified the significant difference (Fig 1 and S1 Table found in the supporting information) [11]. Moreover, starting in 2012, a new allocation system was proposed that used the kidney donor risk index (KDRI) in addition to the EPTS model [12]. Therefore, in the analysis we used data that includes all kidney transplants performed between January 1, 2002 and December 31, 2011. Observations after 2012 would not have a 5-year post-transplant window at the time of this study. 5-year or longer time horizons for kidney transplant survival models have often been used in the literature [6, 12, 13].

There were 163,199 observations available during the chosen ten-year time period with 487 variables. We removed variables not present in more than 95% of the observations unless they were identified as important in the previous literature [14–16]. In the latter case we removed variables not present in more than 80% of the observations. We also removed variables that were recorded twice, or were known only after the kidney transplant. The resulting data set had 73 variables.

The following approaches were used in addressing the issue of missing data: (i) imputation by predictive mean matching (PMM), and (ii) removing missing data for non-categorical variables. In approach (ii), we labeled missing data for categorical variables as ‘unknown’ and removed the non-categorical observations with missing data. Variable selection and all other analysis was carried out using approach (ii), unless specified otherwise. We cross-validated our proposed predictive model using both approaches. When cross-validating our final predictive model with approach (ii), 17% of the data were removed.

#### Grouping categorical variables

In the data, some of the categorical variables have a large number of possible values. For example, the variable kidney diagnosis, has 75 different possible values. To avoid overfitting and large model variance, we used the approach described in S1 Text and illustrated in S2 Table to group different values of the variable together. The values grouped together have a similar effect on the hazard function, controlling for relevant variables. Following this approach, we decreased the number of different kidney diagnosis values from 75 to 8.

## Methods

### Variable selection

For variable selection, we first used the Breiman-Cutler permutation importance measure for random survival forests to rank the variables in order of variable importance [17]. Harrell’s concordance index was used to measure the error rate for assessing the decrease in accuracy when permuting each predictor variable in the permutation importance calculation.

**Fig 2:**
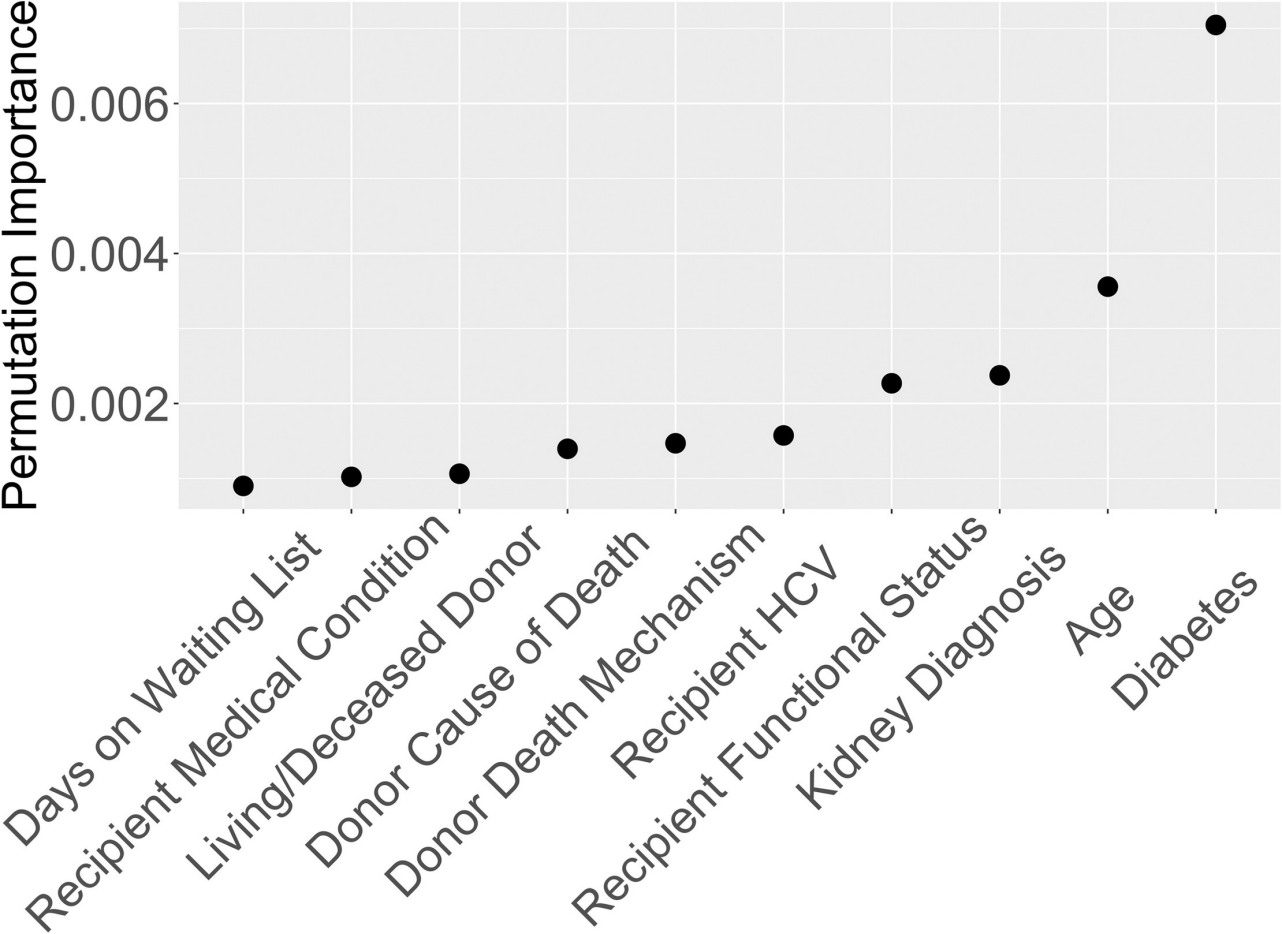
Variable importance for recipients ages 50 and under based on Breiman-Cutler permutation importance.

**Fig 3:**
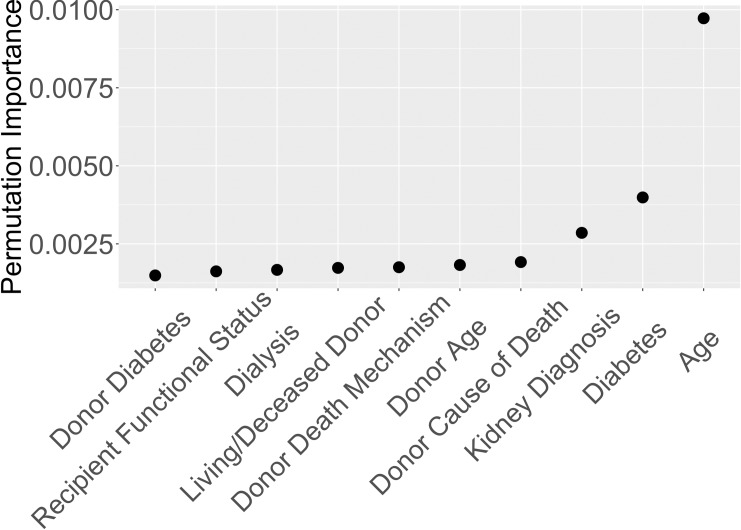
Variable importance for recipients ages 51 and older based on Breiman-Cutler permutation importance.

Recipient age was ranked as the most important variable by permutation importance on the entire dataset. Hence, we decided to split the data into age-based cohorts and produced two separate rankings of variables, one ranking for older recipients and one for younger recipients. This allowed us to build two predictive models for the different cohorts. To find the split value for recipient age, we built 100 survival decision trees [18, 19], each with one split using only recipient age. Each decision tree finds the recipient age that gives the best binary split of two groups based on parameters suggested by Strobl et al. [20]. The average split value for the 100 trees was 48.7 years. Hence, rounding up to 50, we performed variable selection separately for transplant recipients aged 50 and under (cohort 1), and recipients aged 51 and older (cohort 2). The average 5-year survival probabilities are 93% and 80% for cohorts 1 and 2, respectively, based on the Kaplan-Meier [21] estimate. Figs 2 and 3 depict the top ten variables for cohorts 1 and 2, respectively, based on random survival forests permutation importance.

**Fig 4:**
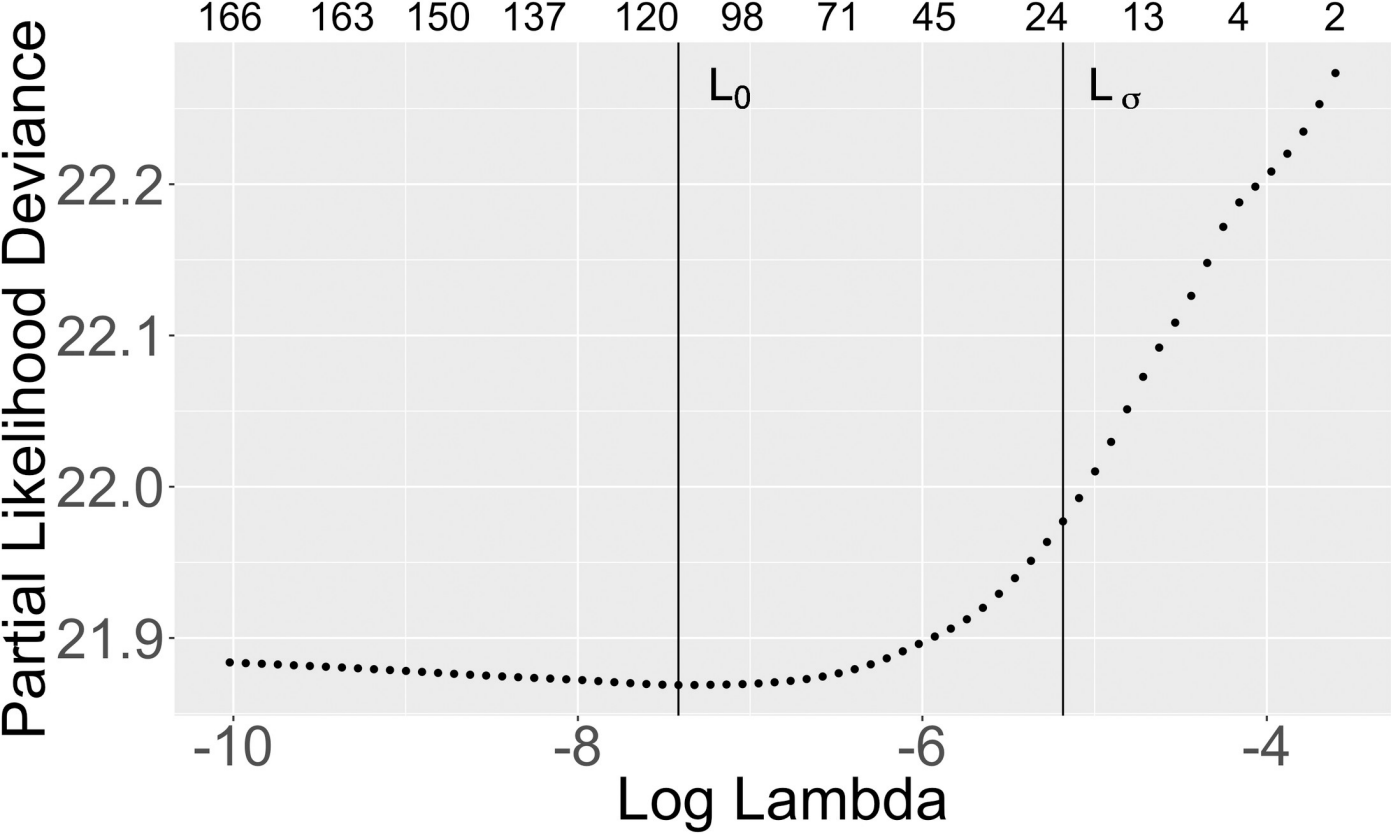
Cox lasso variable selection for recipients ages 50 and under. The top row represents the number of non-zero coefficients per Lasso penalty value, lambda. The vertical line L0 corresponds to the optimal penalty, which minimizes the PLD. The line Lσ corresponds to the largest penalty value corresponding to the PLD values within one standard deviation of the minimum PLD.

We then used a Cox model regularized with the Lasso (L1) penalty to help determine how many of the top variables to select [22]. We used 10-fold cross-validation to determine the optimal Lasso penalty. Fig 4 shows the number of nonzero coefficients for different penalty values for cohort 1. The top row represents the number of non-zero coefficients per different values of the Lasso penalty. The vertical line L0 corresponds to the optimal penalty, which minimizes the Partial Likelihood Deviance (PLD). The line Lσ corresponds to the largest penalty value corresponding to the PLD values within one standard deviation of the minimum PLD. S1 Fig gives the analogous results for cohort 2. To keep the predictive model simple and minimize the number of variables, we used the Lσ penalty, which has fewer nonzero coefficients than using L0.

For each cohort, variables that have nonzero coefficients in the Lasso model and which are also among the top 20 variables chosen by permutation importance are included in the predictive model. Table 1 lists the final selections of variables for each cohort. S3 Table provides a description of the variables we used in our proposed predictive survival model. S4 Table gives the mean values from the data for numeric variables and the percentages of observations for each category for categorical variables.

**Table 1:** Variables in the proposed predictive model.

Cohort 1: Ages 50 and Under	Cohort 2: Ages 51 and Older
AGE	AGE
COLD_ISCH_KI	AGE_DON
CREAT_TRR	ANY_DIAL
DEATH_MECH_DON	COD_CAD_DON
DIAB	COLD_ISCH_KI
DIAG_KI	CREAT_TRR
ETHCAT	DEATH_MECH_DON
FUNC_STAT_TRR	DIAB
HCV_SEROSTATUS	DIAG_KI
HIST_DIABETES_DON	DRUGTRT_COPD
HIST_HYPERTENS_DON	ETHCAT
MED_COND_TRR	FUNC_STAT_TRR
PAYMENTSOURCE_AT_TRANSPLANT	HCV_SEROSTATUS
REGION	HIST_HYPERTENS_DON

A description of each variable is given in S3 Table.

### Predictive models

For cohort 1, we built a random survival forest model with conditional inference trees as base learners [18, 19]. We grew a forest with 800 trees and four randomly selected variables considered for each split. Random forest parameters suggested by Strobl et al. [20] were used with a slight modification. We restricted a tree split to occur only if the splitting test statistic exceeded 0.3, instead of guaranteeing the inclusion of all splits. In testing, we found that this allowed the use of smaller trees with the same predictive performance measured by Harrell’s concordance index.

For cohort 2, the Cox proportional hazards model achieved a better concordance index than using random survival forests (0.664 vs. 0.655 based on 10 cross-validation samples of 80% training data and 20% out-of-sample data). Hence for cohort 2, we fit a Cox proportional hazards model. S5 Table shows the coefficients for the Cox model when it was trained on 100,000 observations.

In the proposed predictive model, we use the combination of random survival forests for cohort 1, and the Cox model for cohort 2. We evaluated the performance of the proposed model compared to other models by two metrics using cross-validation: (i) Harrell’s concordance index, and (ii) the integrated Brier score [23]. In addition to comparing the performance of our model to the reported performance of the EPTS model [2], we evaluated the EPTS model on the same data that we used for our model. We used PMM for missing data because the EPTS model does not allow for variable inputs to be unknown. We validated our proposed model in multiple ways, using PMM imputation and without imputation.

Our methodology for building the proposed predictive model is described by the following high-level summary:

1. Identify important predictive variables by performing variable selection techniques such as Lasso or permutation importance.
2. Test the performance of multiple predictive models on the data using the variables identified in step 1. Use cross-validation and metrics such as the concordance index to evaluate the performance.
3. Determine the best binary split in the data using methods such as decision trees.
4. Repeat steps 1–3 for both subsets of the data a specified number of times. The final model consists of combining the predictions from the models that perform best on the different subsets of the data.

## Results

Table 2 shows the 5-year Harrell’s concordance index and the integrated Brier score for the prosed model using 10 random samples of 80,000 training observations and 20,000 out-of-sample observations. It also reports the performance of a number of other models from the recent literature. Harrell’s 5-year concordance index for our proposed model is 0.724 versus 0.69 reported for the EPTS model [2] and 0.697 for the EPTS model applied to the data used for this study. The concordance index of the proposed model is 0.717 when we remove the donor variables and include only the recipient variables. This provides a more direct comparison to the EPTS model since the EPTS model does not use donor variables. The performance of the proposed model was nearly the same when we also validated it using PMM imputation as opposed to validation without imputation.

**Table 2:** Performance of the proposed predictive model compared to other models.

Model	5-Year C-index	5-Year Integrated Brier Score
LYFT Reported [4]	0.680	Not Reported
EPTS Reported [2]	0.69	Not Reported
EPTS Using the Same Cross-Validation Data as the Proposed Model	0.697	Not Calculated
Li et al. [6] Reported	0.700	Not Reported
Cox Model for Both Cohorts*	0.706	0.063
Random Forests for Both Cohorts*	0.718	0.062
Proposed Model without Donor Variables**	0.717	0.060
Proposed Model	0.724	0.061
Proposed Model using PMM Imputation	0.724	0.060

Performance from 10 random samples of 80,000 training observations and 20,000 out-of-sample observations for all models except those marked ‘Reported’, where the metrics shown were provided in the literature for their respective models.

*These two predictive models used the variable selection techniques we used for each cohort separately but instead applied to all the data.

**Donor variables that were removed were: AGE_DON, COD_CAD_DON, COLD_ISCH_KI, DEATH_MECH_DON, HIST_DIABETES_DON, and HIST_HYPERTENS_DON.

**Fig 5:**
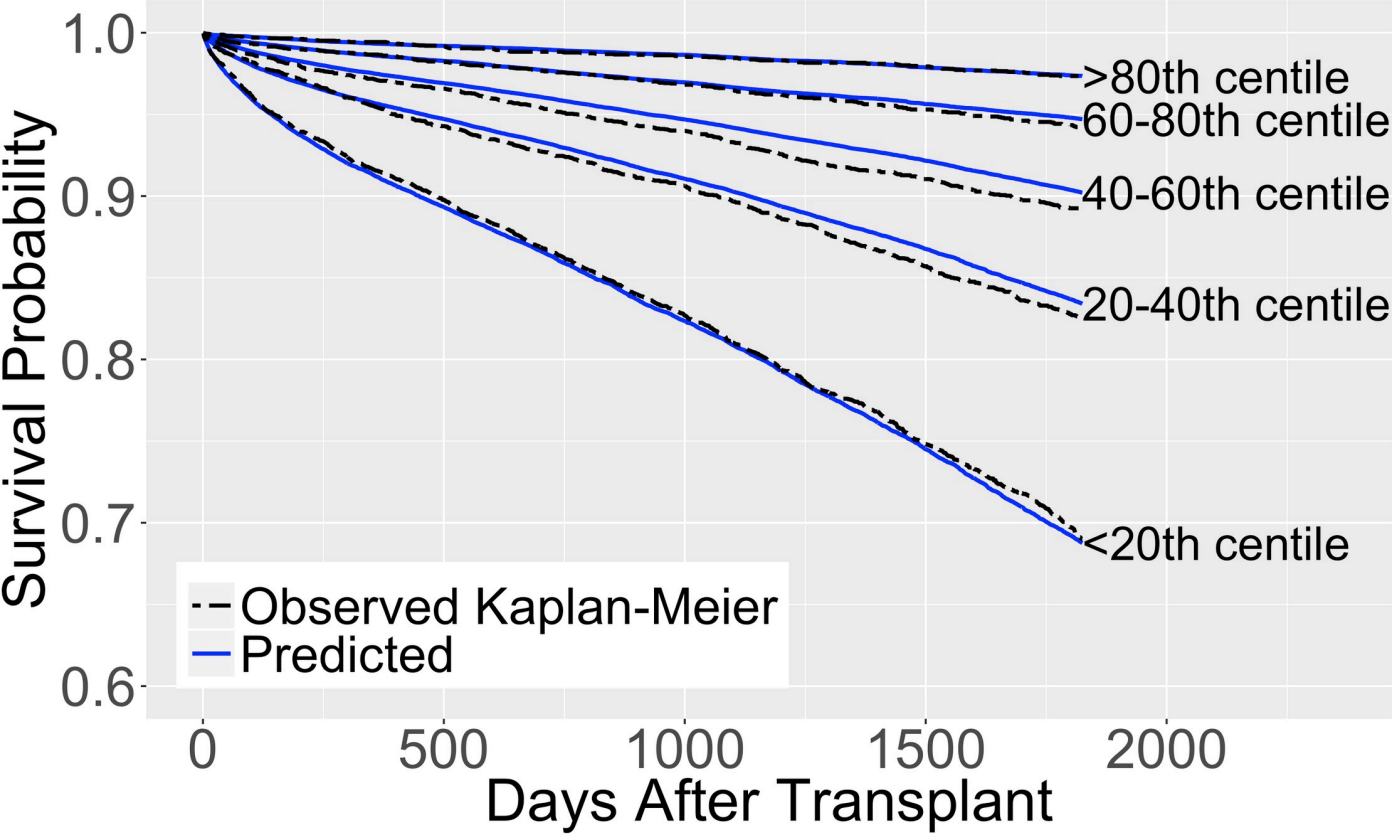
Predicted survival of the proposed model. Trained on 100,000 observations and validated on 25,000 out-of-sample observations. The survival curves are separated into 5 groups based on the predicted 5-year survival in the out-of-sample data.

**Fig 6:**
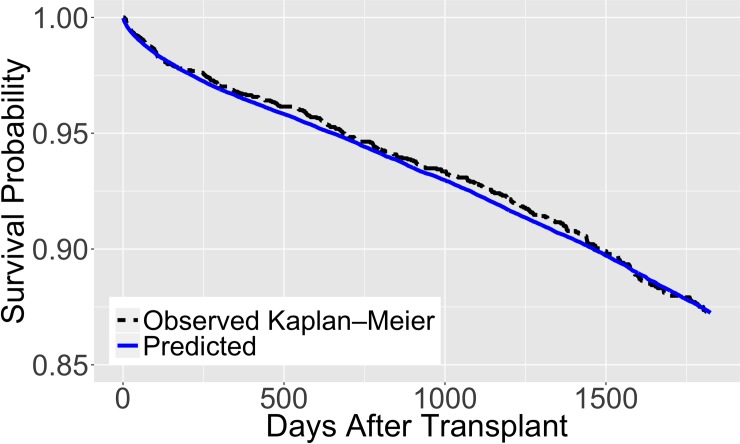
Predicted survival of the proposed model for a ‘typical’ kidney transplant recipient. Trained on 100,000 observations and validated on 25,000 out-of-sample observations. In the out-of-sample data, an observation is considered ‘typical’ if the values are within one standard deviation of the mean for recipient age, donor age, and cold ischemia time, and the most common values from the data for recipient diabetes, recipient dialysis status, recipient medical condition, and donor hypertension status.

Figs 5 and 6 illustrate the behavior of our proposed model trained using a random sample of 100,000 observations and validated on 25,000 out-of-sample observations. The solid lines represent the survival predictions and the dotted lines depict the observed Kaplan-Meier estimates for the out-of-sample observations. We also illustrate the model’s survival predictions for different values of its variables, holding the remaining variables constant, in S2 Fig (also see S6 and S7 Tables). S8 and S9 Tables present results on the performance of our proposed model at different numbers of days after transplantation and on different categories respectively. S10 Table gives results for additional tests on the performance of our proposed model and the EPTS model on data without pediatric recipients and living donors. S11 Table shows the performance of the proposed model compared to the EPTS model on the general population for each cross-validation sample.

## Discussion

The current kidney allocation system, adopted in 2013, matches the best 20% of kidneys as determined by the KDRI, to the top 20% of potential recipients with the highest predicted transplant survival probabilities, estimated by the EPTS model [12]. Improving the predictive performance of kidney transplant survival models can help the kidney allocation system more accurately rank potential recipients based on estimates of post-transplant survival. Table 2 shows that our proposed model has a higher concordance index than the EPTS model and other models recently published in the literature, such as the LYFT model and the flexible parametric model proposed by Li et al. [6]. Hence, when considering a random pair of candidates and determining which candidate in the pair has a higher post-transplant survival probability (in a given time frame, e.g., 5 years), our model will result in more correct pair rankings than the EPTS model, and has the potential to significantly improve the matching of organs to recipients. We also found that by using a model that combines different predictive models and variables for different age groups, we achieved better performance than by using the same model and variables for both cohorts.

A comparison of kidney transplant survival models over time shows a concordance index of 0.68 in 2009 for the LYFT model (Wolfe et al., 2009), a concordance index of 0.69 in 2013 for the EPTS model currently used in the kidney allocation system (Clayton et al., 2014), and an index of 0.70 in 2016 from Li et al. (2016). A gain in the index of 0.01 can have a dramatic impact considering that these models are used in the U.S. kidney allocation system, which is responsible for allocating tens of thousands of kidneys per year. Our model yields an improvement in the concordance index of 0.03 over the EPTS model when tested on the same data, which can have a significant impact on ranking kidney waitlist patients by their post-transplant survival more accurately.

Unlike the EPTS model, the proposed model has the flexibility to take into account characteristics of donors; hence, it can help predict which donor-recipient matching can result in the highest post-transplant survival, among several choices. The proposed model can also be used without the donor variables, and still result in a better concordance index than the EPTS model. This can be useful in estimating the survival of transplant recipients before the donor information is known, such as when determining recipient priority in the allocation system.

Our findings suggest that there may be a benefit for building separate models for different cohorts of patients (in this case cohorts separated by recipient age). For example, the variable region, was in the top ten ranked variables for cohort 1 but not for cohort 2. Hence, the impact of region on post-transplant survival is higher for cohort 1 than cohort 2.

The proposed model uses machine learning methods, and although it does not result in a simple equation to predict transplant survival, such methods are straightforward to apply. Further, the proposed model uses 18 different predictive variables versus 4 used by EPTS. While the model complexity could be viewed as a limitation, with the increasing power of computer tools, the model can easily be implemented in practice.

## Supporting information

S1 Table Log-Rank test for differences in cohort survival. χ2 statistic value = 259.3, *p*-value = 0.
(DOCX)

S2 Table Original kidney diagnosis values and their new groupings. We used the coefficients of a Cox model to group values with similar predicted transplant survival, controlling for other variables. We controlled for: recipient age, recipient diabetes, recipient cold ischemia time, recipient initial waitlist status, recipient ethnicity, donor age, donor cause of death, and donor living status. We considered putting the first three values of kidney diagnosis in S2 Table in its own group. However, they had fewer than 90 observations combined, which may cause overfitting issues.
(DOCX)

S3 Table Descriptions of variables used in the proposed model. *See S2 Table for variable values in this group.
(DOCX)

S4 Table Summary of variables used in the predictive models. The mean value of the data is given for numeric variables and the percentage of observations for each category is given for categorical variables. *See S2 Table for variable values in this group.
(DOCX)

S5 Table Cox proportional hazards model coefficients for the proposed model. Trained on a Random Sample of 100,000 observations.
(DOCX)

S6 Table Variables held constant in S2 Fig. *See S2 Table for variable values in this group.
(DOCX)

S7 Table Factor level legend for S2 Fig. *See S2 Table for variable values in this group.
(DOCX)

S8 Table Concordance index at different days after transplant for the proposed model. The performance is calculated from 10 random samples of 80,000 training observations and 20,000 out-of-sample observations.
(DOCX)

S9 Table Proposed model performance by category. Performance from a random sample of 100,000 training observations and 25,000 out-of-sample observations.
(DOCX)

S10 Table Additional model testing. 10 random samples of 80,000 training observations and 20,000 out-of-sample observations. **Donor variables that were removed: AGE_DON, COD_CAD_DON, COLD_ISCH_KI, DEATH_MECH_DON, HIST_DIABETES_DON, and HIST_HYPERTENS_DON.
(DOCX)

S11 Table Proposed model and EPTS model cross-validation results in Table 2. C-index based on 10 random samples of 80,000 training observations and 20,000 out-of-sample observations. Using a paired two sample Student's t-test, we reject the null hypothesis that the difference in model performance means is equal to 0 (p-value = 2.4x10^-11^). We also used a Shapiro-Wilk normality test and an F test for equality of variances to verify the assumptions of the t-test (Shapiro-Wilk p-value of 0.654 and 0.298 for the proposed model data and EPTS model data respectively; hence we don’t reject the null hypothesis of normally distributed model performance results. F test p-value of 0.605; hence we don’t reject the null hypothesis of equality of model performance variance).
(DOCX)

S1 Text Grouping values for the variable kidney diagnosis. (DOCX)

S2 Text Software. The analysis was undertaken using the statistical software R version 3.3.2 as well as several key packages listed in the references [24–30].
(DOCX)

S1 Fig Lasso variable selection for recipients ages 51 and older. (TIF)

S2 Fig Proposed model’s 5-year survival predictions for different variable values. In each plot, variable values not shown are held constant and listed in S6 Table. The model was trained on 100,000 random training observations.
(TIF)
