# Supplementary material for: Using machine learning and an ensemble of methods to predict kidney transplant survival
Source: PLoS One. 2019 Jan 9;14(1):e0209068. doi: 10.1371/journal.pone.0209068 (PMC6326487; doi:10.1371/journal.pone.0209068)
Supplement: S1 Table — χ2 statistic value = 259.3, p-value = 0. (DOCX) [file pone.0209068.s001.docx]

**S1 Table. Log-Rank Test for Differences in Cohort Survival.**

| **Cohort** | **Observed** | **Expected** |
| --- | --- | --- |
| 1987–2001 | 47915 | 45945 |
| 2002–2014 | 25860 | 27830 |

χ2 statistic value = 259.3, *p*-value = 0.
