# Supplementary material for: Using machine learning and an ensemble of methods to predict kidney transplant survival
Source: PLoS One. 2019 Jan 9;14(1):e0209068. doi: 10.1371/journal.pone.0209068 (PMC6326487; doi:10.1371/journal.pone.0209068)
Supplement: S2 Table — We used the coefficients of a Cox model to group values with similar predicted transplant survival, controlling for other variables. We controlled for: recipient age, recipient diabetes, recipient cold ischemia time, recipient initial waitlist status, recipient ethnicity, donor age, donor cause of death, and donor living status. We considered putting the first three values of kidney diagnosis in S2 Table in its own group. However, they had fewer than 90 observations combined, which may cause overfitting issues. (DOCX) [file pone.0209068.s002.docx]

**S2 Table. Original Kidney Diagnosis Values and Their New Groupings.**

| **Factor Level** | **Coefficient** | **New Group** |
| --- | --- | --- |
| THIN BASEMENT MEMBRANE DISEASE | -12.018 | 1 |
| HIV NEPHROPATHY | -11.659 | 1 |
| DYSPLASIA | -10.185 | 1 |
| GOUT | -1.655 | 1 |
| MEDULLARY CYSTIC DISEASE | -1.042 | 1 |
| GOODPASTURE'S SYNDROME | -0.771 | 1 |
| LYMPHOMA | -0.732 | 1 |
| ALPORT'S SYNDROME | -0.689 | 1 |
| IGA NEPHROPATHY | -0.629 | 1 |
| ANTI-GBM | -0.585 | 2 |
| FAMILIAL NEPHROPATHY | -0.572 | 2 |
| POLYCYSTIC KIDNEYS | -0.551 | 2 |
| RHEUMATOID ARTHRITIS | -0.518 | 2 |
| HENOCH-SCHOENLEIN PURPURA | -0.416 | 2 |
| FOCAL GLOMERULAR SCLEROSIS (FOCAL SEGMENTAL - FSG) | -0.287 | 2 |
| CHRONIC PYELONEPHRITIS/REFLUX NEPHROPATH | -0.217 | 2 |
| UROLITHIASIS | -0.210 | 2 |
| IDIO/POST-INF CRESCENTIC GLOMERULONEPHRI | -0.209 | 3 |
| CONGENITAL OBSTRUCTIVE UROPATHY | -0.205 | 3 |
| NEPHROLITHIASIS | -0.201 | 3 |
| CHRONIC GLOMERULONEPHRITIS UNSPECIFIED | -0.196 | 3 |
| MEMBRANOUS GLOMERULONEPHRITIS | -0.160 | 3 |
| RAPID PROGRESSIVE GLOMERULONEPHRITIS (RPGN) | -0.094 | 3 |
| WEGENERS GRANULOMATOSIS | -0.092 | 3 |
| MALIGNANT HYPERTENSION | -0.077 | 3 |
| MEMBRANOUS NEPHROPATHY | -0.060 | 3 |
| CHRONIC NEPHROSCLEROSIS-UNSPECIFIED | -0.054 | 4 |
| DRUG RELATED INTERSTITIAL NEPHRITIS | -0.022 | 4 |
| CHRONIC GLOMERULOSCLEROSIS UNSPECIFIED | -0.019 | 4 |
| NEPHRITIS | -0.009 | 4 |
| ACQUIRED OBSTRUCTIVE NEPHROPATHY | 0.000 | 4 |
| OXALATE NEPHROPATHY (INCLUDES HEREDITARY OXALOSIS) | 0.025 | 4 |
| HYPERTENSIVE NEPHROSCLEROSIS | 0.038 | 4 |
| SYSTEMIC LUPUS ERYTHEMATOSUS | 0.041 | 4 |
| RENAL CELL CARCINOMA | 0.062 | 5 |
| NEPHROPHTHISIS | 0.064 | 5 |
| MESANGIO-CAPILLARY 1 GLOMERULONEPHRITIS | 0.121 | 5 |
| CHOLESTEROL EMBOLIZATION | 0.125 | 5 |
| POLYARTERITIS | 0.137 | 5 |
| OTHER SPECIFY | 0.141 | 5 |
| RENAL ARTERY THROMBOSIS | 0.142 | 5 |
| ANTIBIOTIC-INDUCED NEPHRITIS | 0.152 | 5 |
| SARCOIDOSIS | 0.153 | 6 |
| HYPOPLASIA/DYSPLASIA/DYSGENSIS/AGENESIS | 0.166 | 6 |
| HEROIN NEPHROTOXICITY | 0.181 | 6 |
| DIABETES | 0.182 | 6 |
| ANALGESIC NEPHROPATHY | 0.194 | 6 |
| INCIDENTAL CARCINOMA | 0.203 | 6 |
| HEMOLYTIC UREMIC SYNDROME | 0.211 | 6 |
| RETRANSPLANT/GRAFT FAILURE | 0.218 | 6 |
| PROGRESSIVE SYSTEMIC SCLEROSIS | 0.252 | 6 |
| PRUNE BELLY SYNDROME | 0.314 | 7 |
| ACUTE TUBULAR NECROSIS | 0.373 | 7 |
| FABRY'S DISEASE | 0.384 | 7 |
| CANCER CHEMOTHERAPY INDUCED NEPHRITIS | 0.424 | 7 |
| MYELOMA | 0.446 | 7 |
| WILMS' TUMOR | 0.479 | 7 |
| CORTICAL NECROSIS | 0.486 | 7 |
| MESANGIO-CAPILLARY 2 GLOMERULONEPHRITIS | 0.491 | 7 |
| LITHIUM TOXICITY | 0.506 | 8 |
| AMYLOIDOSIS | 0.595 | 8 |
| SCLERODERMA | 0.611 | 8 |
| CALCINEURIN INHIBITOR NEPHROTOXICITY | 0.617 | 8 |
| CYSTINOSIS | 0.685 | 8 |
| RADIATION NEPHRITIS | 0.740 | 8 |
| PRE-BMTRANSPLANTATION TOTAL BODY IRRADIATION | 0.748 | 8 |
| HEPATORENAL SYNDROME | 0.765 | 8 |
| SICKLE CELL ANEMIA | 1.034 | 8 |

We used the coefficients of a Cox model to group values with similar predicted transplant survival, controlling for other variables. We controlled for: recipient age, recipient diabetes, recipient cold ischemia time, recipient initial waitlist status, recipient ethnicity, donor age, donor cause of death, and donor living status. We considered putting the first three values of kidney diagnosis in S2 Table in its own group. However, they had fewer than 90 observations combined, which may cause overfitting issues.
