# Supplementary material for: Using machine learning and an ensemble of methods to predict kidney transplant survival
Source: PLoS One. 2019 Jan 9;14(1):e0209068. doi: 10.1371/journal.pone.0209068 (PMC6326487; doi:10.1371/journal.pone.0209068)
Supplement: S3 Table — *See S2 Table for variable values in this group. (DOCX) [file pone.0209068.s003.docx]

**S3 Table. Descriptions of Variables Used in the Proposed Model.**

| **Variable Name** | **Description** | **Categories** |
| --- | --- | --- |
| AGE | Recipient age (yrs) | NA |
| AGE_DON | Donor age (yrs) | NA |
| ANY_DIAL | Recipient on dialysis any time between registration and transplant | NO, NOT_KNOWN, YES |
| COD_CAD_DON | Deceased donor-cause of death | ANOXIA, CEREBROVASCULAR/STROKE, CNS TUMOR, HEAD TRAUMA, NOT_KNOWN, OTHER SPECIFY |
| COLD_ISCH_KI | Kidney cold ischemic time (hours) | NA |
| CREAT_TRR | Recipient serum creatinine at time of transplant | NA |
| DEATH_MECH_DON | Deceased donor-mechanism of death | ASPHYXIATION, BLUNT INJURY, CARDIOVASCULAR, DEATH FROM NATURAL CAUSES, DROWNING, DRUG INTOXICATION, ELECTRICAL, INTRACRANIAL HEMORRHAGE/STROKE, NONE OF THE ABOVE, NOT_KNOWN, SEIZURE, SIDS, STAB OR GUNSHOT WOUND |
| DIAB | Recipient diabetes at registration | NO, NOT_KNOWN, YES |
| DIAG_KI | Kidney recipient primary diagnosis at transplant | GROUP_1*, GROUP_2*, GROUP_3*, GROUP_4*, GROUP_5*, GROUP_6*, GROUP_7*, GROUP_8*, NOT_KNOWN |
| DRUGTRT_COPD | Recipient drug treated COPD at registration | NO, NOT_KNOWN, YES |
| ETHCAT | Recipient ethnicity category | AMER IND/ALASKA NATIVE, ASIAN, BLACK, HISPANIC, MULTIRACIAL, NATIVE HAWAIIAN/OTHER PACIFIC ISLANDER, NOT_KNOWN, WHITE |
| FUNC_STAT_TRR | Recipient functional status at transplant | 10-20 PERCENT VERY SICK HOSPITALIZATION NECESSARY, 30-50 PERCENT REQUIRES CONSIDERABLE ASSISTANCE BUT DEATH NOT IMMINENT, 60-70 PERCENT PERFORMS ACTIVITIES OF DAILY LIVING WITH SOME ASSISTANCE, 80-100 PERCENT PERFORMS ACTIVITIES OF DAILY LIVING WITH NO ASSISTANCE, NOT APPLICABLE (PATIENT < 1 YEAR OLD), NOT_KNOWN, PERFORMS ACTIVITIES OF DAILY LIVING WITH TOTAL ASSISTANCE. |
| HCV_SEROSTATUS | Recipient HCV status | NEGATIVE, NOT DONE, NOT_KNOWN, POSITIVE |
| HIST_DIABETES_DON | Deceased donor-history of diabetes, including duration of disease | NO, NOT_KNOWN, YES |
| HIST_HYPERTENS_DON | Deceased donor-history of hypertension | NO, NOT_KNOWN, YES |
| MED_COND_TRR | Recipient medical condition pre-transplant at transplant | HOSPITALIZED NOT IN ICU, IN INTENSIVE CARE UNIT, NOT HOSPITALIZED |
| PAYMENTSOURCE_AT_TRANSPLANT | Recipient primary payment source | CHIP, DONATION OR FREE CARE, MEDICAID, MEDICARE, NOT_KNOWN, OTHER, OTHER GOVERNMENT OR DEPARTMENT OF VA, SELF, SOME PRIVATE BY PRIMARY OR SECONDARY |
| REGION | UNOS region where transplanted | 1, 2, 3, 4, 5, 6, 7, 8, 9, 10, 11 |

*See S2 Table for variable values in this group.
