# Supplementary material for: Using machine learning and an ensemble of methods to predict kidney transplant survival
Source: PLoS One. 2019 Jan 9;14(1):e0209068. doi: 10.1371/journal.pone.0209068 (PMC6326487; doi:10.1371/journal.pone.0209068)
Supplement: S4 Table — The mean value of the data is given for numeric variables and the percentage of observations for each category is given for categorical variables. *See S2 Table for variable values in this group. (DOCX) [file pone.0209068.s004.docx]

**S4 Table. Summary of Variables Used in the Predictive Models.**

| **Variable** | **Variable Summary** |
| --- | --- |
| AGE | 48.5 |
| AGE_DON | 38.7 |
| ANY_DIAL: N | 6% |
| ANY_DIAL: NOT KNOWN | 11.3% |
| ANY_DIAL: Y | 82.6% |
| COD_CAD_DON: ANOXIA | 12.9% |
| COD_CAD_DON: CEREBROVASCULAR/STROKE | 24.8% |
| COD_CAD_DON: CNS TUMOR | 0.4% |
| COD_CAD_DON: HEAD TRAUMA | 28.1% |
| COD_CAD_DON: NOT KNOWN | 32.3% |
| COD_CAD_DON: OTHER SPECIFY | 1.6% |
| COLD_ISCH_KI | 12.8 |
| CREAT_TRR | 7.8 |
| DEATH_MECH_DON: ASPHYXIATION | 2.1% |
| DEATH_MECH_DON: BLUNT INJURY | 19.1% |
| DEATH_MECH_DON: CARDIOVASCULAR | 6.5% |
| DEATH_MECH_DON: DEATH FROM NATURAL CAUSES | 1.1% |
| DEATH_MECH_DON: DROWNING | 0.6% |
| DEATH_MECH_DON: DRUG INTOXICATION | 2.4% |
| DEATH_MECH_DON: ELECTRICAL | <0.1% |
| DEATH_MECH_DON: INTRACRANIAL HEMORRHAGE/STROKE | 25.9% |
| DEATH_MECH_DON: NONE OF THE ABOVE | 2% |
| DEATH_MECH_DON: NOT KNOWN | 32.3% |
| DEATH_MECH_DON: SEIZURE | 0.6% |
| DEATH_MECH_DON: SIDS | <0.1% |
| DEATH_MECH_DON: STAB OR GUNSHOT WOUND | 7.3% |
| DIAB: NO | 68.5% |
| DIAB: NOT KNOWN | 1.5% |
| DIAB: YES | 30% |
| DIAG_KI: GROUP_1* | 5.6% |
| DIAG_KI: GROUP_2* | 16.9% |
| DIAG_KI: GROUP_3* | 10.5% |
| DIAG_KI: GROUP_4* | 25.3% |
| DIAG_KI: GROUP_5* | 7.3% |
| DIAG_KI: GROUP_6* | 31.6% |
| DIAG_KI: GROUP_7* | 0.7% |
| DIAG_KI: GROUP_8* | 1.5% |
| DIAG_KI: NOT KNOWN | 0.7% |
| DRUGTRT_COPD: N | 94.7% |
| DRUGTRT_COPD: NOT KNOWN | 4.3% |
| DRUGTRT_COPD: Y | 1% |
| ETHCAT: AMER IND/ALASKA NATIVE | 0.9% |
| ETHCAT: ASIAN | 5% |
| ETHCAT: BLACK | 25.6% |
| ETHCAT: HISPANIC | 14% |
| ETHCAT: MULTIRACIAL | 0.6% |
| ETHCAT: NATIVE HAWAIIAN/OTHER PACIFIC ISLANDER | 0.4% |
| ETHCAT: NOT KNOWN | <0.1% |
| ETHCAT: WHITE | 53.5% |
| FUNC_STAT_TRR: 10-20 PERCENT VERY SICK HOSPITALIZATION NECESSARY | 0.5% |
| FUNC_STAT_TRR: 30-50 PERCENT REQUIRES CONSIDERABLE ASSISTANCE BUT DEATH NOT IMMINENT | 2.7% |
| FUNC_STAT_TRR: 60-70 PERCENT PERFORMS ACTIVITIES OF DAILY LIVING WITH SOME ASSISTANCE | 17.7% |
| FUNC_STAT_TRR: 80-100 PERCENT PERFORMS ACTIVITIES OF DAILY LIVING WITH NO ASSISTANCE | 73.3% |
| FUNC_STAT_TRR: NOT APPLICABLE (PATIENT < 1 YEAR OLD) | 0.5% |
| FUNC_STAT_TRR: NOT KNOWN | 5.2% |
| FUNC_STAT_TRR: PERFORMS ACTIVITIES OF DAILY LIVING WITH TOTAL ASSISTANCE. | 0.1% |
| HCV_SEROSTATUS: NEGATIVE | 87.6% |
| HCV_SEROSTATUS: NOT DONE | 4.2% |
| HCV_SEROSTATUS: NOT KNOWN | 3.2% |
| HCV_SEROSTATUS: POSITIVE | 5.1% |
| HIST_DIABETES_DON: NO | 63.2% |
| HIST_DIABETES_DON: NOT KNOWN | 32.6% |
| HIST_DIABETES_DON: YES | 4.2% |
| HIST_HYPERTENS_DON: N | 75.4% |
| HIST_HYPERTENS_DON: NOT KNOWN | 7% |
| HIST_HYPERTENS_DON: Y | 17.7% |
| MED_COND_TRR: HOSPITALIZED NOT IN ICU | 1.7% |
| MED_COND_TRR: IN INTENSIVE CARE UNIT | 0.6% |
| MED_COND_TRR: NOT HOSPITALIZED | 97.8% |
| PAYMENTSOURCE_AT_TRANSPLANT: CHIP | 0.2% |
| PAYMENTSOURCE_AT_TRANSPLANT: DONATION OR FREE CARE | 0.1% |
| PAYMENTSOURCE_AT_TRANSPLANT: MEDICAID | 5.4% |
| PAYMENTSOURCE_AT_TRANSPLANT: MEDICARE | 44.5% |
| PAYMENTSOURCE_AT_TRANSPLANT: NOT KNOWN | <0.1% |
| PAYMENTSOURCE_AT_TRANSPLANT: OTHER | 0.1% |
| PAYMENTSOURCE_AT_TRANSPLANT: OTHER GOVERNMENT OR DEPARTMENT OF VA | 1.4% |
| PAYMENTSOURCE_AT_TRANSPLANT: SELF | 0.2% |
| PAYMENTSOURCE_AT_TRANSPLANT: SOME PRIVATE BY PRIMARY OR SECONDARY | 48.2% |
| REGION: 1 | 3.1% |
| REGION: 10 | 9.3% |
| REGION: 11 | 9.6% |
| REGION: 2 | 14.1% |
| REGION: 3 | 12.6% |
| REGION: 4 | 8.2% |
| REGION: 5 | 15.8% |
| REGION: 6 | 3.8% |
| REGION: 7 | 9.7% |
| REGION: 8 | 6.9% |
| REGION: 9 | 7% |

The mean value of the data is given for numeric variables and the percentage of observations for each category is given for categorical variables. *See S2 Table for variable values in this group.
