# Supplementary material for: Using machine learning and an ensemble of methods to predict kidney transplant survival
Source: PLoS One. 2019 Jan 9;14(1):e0209068. doi: 10.1371/journal.pone.0209068 (PMC6326487; doi:10.1371/journal.pone.0209068)
Supplement: S5 Table — Trained on a Random Sample of 100,000 observations. (DOCX) [file pone.0209068.s005.docx]

**S5 Table. Cox Proportional Hazards Model Coefficients for the Proposed Model.**

| **Variable Name** | **Coefficient** | ***p*-Value** |
| --- | --- | --- |
| AGE | 0.05 | 0 |
| AGE_DON | 0.01 | 0 |
| ANY_DIAL: Base Level - NO |  |  |
| ANY_DIAL: NOT_KNOWN | -0.05 | 0.37 |
| ANY_DIAL: YES | 0.45 | 0 |
| COD_CAD_DON: Base Level - ANOXIA |  |  |
| COD_CAD_DON: CEREBROVASCULAR STROKE | -0.08 | 0.21 |
| COD_CAD_DON: CNS TUMOR | -0.21 | 0.15 |
| COD_CAD_DON: HEAD TRAUMA | -0.01 | 0.85 |
| COD_CAD_DON: NOT_KNOWN | -0.35 | 0 |
| COD_CAD_DON: OTHER SPECIFY | -0.13 | 0.12 |
| COLD_ISCH_KI | 0 | 0 |
| CREAT_TRR | -0.03 | 0 |
| DEATH_MECH_DON: Base Level - ASPHYXIATION |  |  |
| DEATH_MECH_DON: BLUNT INJURY | 0 | 0.98 |
| DEATH_MECH_DON: CARDIOVASCULAR | 0.07 | 0.38 |
| DEATH_MECH_DON: DEATH FROM NATURAL CAUSES | 0.3 | 0.01 |
| DEATH_MECH_DON: DROWNING | 0.09 | 0.55 |
| DEATH_MECH_DON: DRUG INTOXICATION | -0.02 | 0.8 |
| DEATH_MECH_DON: ELECTRICAL | -0.98 | 0.17 |
| DEATH_MECH_DON: INTRACRANIAL HEMORRHAGE STROKE | 0.16 | 0.11 |
| DEATH_MECH_DON: NONE OF THE ABOVE | 0.16 | 0.12 |
| DEATH_MECH_DON: SEIZURE | 0.22 | 0.12 |
| DEATH_MECH_DON: SIDS | -0.64 | 0.52 |
| DEATH_MECH_DON: STAB OR GUNSHOT WOUND | -0.01 | 0.9 |
| DIAB: Base Level - NO |  |  |
| DIAB: NOT_KNOWN | -0.01 | 0.88 |
| DIAB: YES | 0.28 | 0 |
| DIAG_KI: Base Level - GROUP_1 |  |  |
| DIAG_KI: GROUP_2 | 0.28 | 0 |
| DIAG_KI: GROUP_3 | 0.5 | 0 |
| DIAG_KI: GROUP_4 | 0.7 | 0 |
| DIAG_KI: GROUP_5 | 0.69 | 0 |
| DIAG_KI: GROUP_6 | 0.79 | 0 |
| DIAG_KI: GROUP_7 | 0.79 | 0 |
| DIAG_KI: GROUP_8 | 1.05 | 0 |
| DIAG_KI: NOT_KNOWN | 0.61 | 0 |
| DRUGTRT_COPD: Base Level - NO |  |  |
| DRUGTRT_COPD: NOT_KNOWN | 0.04 | 0.42 |
| DRUGTRT_COPD: YES | 0.33 | 0 |
| ETHCAT: Base Level - AMER IND/ALASKA NATIVE |  |  |
| ETHCAT: ASIAN | -0.29 | 0 |
| ETHCAT: BLACK | -0.01 | 0.93 |
| ETHCAT: HISPANIC | -0.18 | 0.06 |
| ETHCAT: MULTIRACIAL | 0.17 | 0.28 |
| ETHCAT: NATIVE HAWAIIAN OTHER PACIFIC ISLANDER | -0.02 | 0.92 |
| ETHCAT: NOT_KNOWN | 3.58 | 0 |
| ETHCAT: WHITE | 0.14 | 0.12 |
| FUNC_STAT_TRR: Base Level - 10-20 PERCENT VERY SICK HOSPITALIZATION NECESSARY |  |  |
| FUNC_STAT_TRR: 30 50 PERCENT REQUIRES CONSIDERABLE ASSISTANCE BUT DEATH NOT IMMINENT | -0.6 | 0 |
| FUNC_STAT_TRR: 60 70 PERCENT PERFORMS ACTIVITIES OF DAILY LIVING WITH SOME ASSISTANCE | -0.81 | 0 |
| FUNC_STAT_TRR: 80 100 PERCENT PERFORMS ACTIVITIES OF DAILY LIVING WITH NO ASSISTANCE | -1.03 | 0 |
| FUNC_STAT_TRR: NOT APPLICABLE PATIENT 1 YEAR OLD | -0.56 | 0 |
| FUNC_STAT_TRR: NOT_KNOWN | -0.94 | 0 |
| FUNC_STAT_TRR: PERFORMS ACTIVITIES OF DAILY LIVING WITH TOTAL ASSISTANCE | -0.63 | 0.06 |
| HCV_SEROSTATUS: Base Level - NEGATIVE |  |  |
| HCV_SEROSTATUS: NOT DONE | 0.03 | 0.47 |
| HCV_SEROSTATUS: NOT_KNOWN | 0.07 | 0.13 |
| HCV_SEROSTATUS: POSITIVE | 0.52 | 0 |
| HIST_HYPERTENS_DON: Base Level - NO |  |  |
| HIST_HYPERTENS_DON: NOT_KNOWN | 0.22 | 0 |
| HIST_HYPERTENS_DON: YES | 0.1 | 0 |

Trained on a Random Sample of 100,000 observations.
