# Supplementary material for: Using machine learning and an ensemble of methods to predict kidney transplant survival
Source: PLoS One. 2019 Jan 9;14(1):e0209068. doi: 10.1371/journal.pone.0209068 (PMC6326487; doi:10.1371/journal.pone.0209068)
Supplement: S6 Table — *See S2 Table for variable values in this group. (DOCX) [file pone.0209068.s006.docx]

**S6 Table. Variables Held Constant in S2 Fig.**

| **Constant Features** | **Value** |
| --- | --- |
| AGE | 48.5 |
| AGE_DON | 38.7 |
| ANY_DIAL | Y |
| COD_CAD_DON | NOT_KNOWN |
| COLD_ISCH_KI | 12.8 |
| CREAT_TRR | 7.8 |
| DEATH_MECH_DON | NOT_KNOWN |
| DIAB | NO |
| DIAG_KI | GROUP_6* |
| DRUGTRT_COPD | N |
| ETHCAT | WHITE |
| FUNC_STAT_TRR | 80-100 PERCENT PERFORMS ACTIVITIES OF DAILY LIVING WITH NO ASSISTANCE |
| HCV_SEROSTATUS | NEGATIVE |
| HIST_DIABETES_DON | NO |
| HIST_HYPERTENS_DON | N |
| MED_COND_TRR | NOT HOSPITALIZED |
| PAYMENTSOURCE_AT_TRANSPLANT | SOME PRIVATE BY PRIMARY OR SECONDARY |
| REGION | 5 |

*See S2 Table for variable values in this group.
