# Supplementary material for: Using machine learning and an ensemble of methods to predict kidney transplant survival
Source: PLoS One. 2019 Jan 9;14(1):e0209068. doi: 10.1371/journal.pone.0209068 (PMC6326487; doi:10.1371/journal.pone.0209068)
Supplement: S7 Table — *See S2 Table for variable values in this group. (DOCX) [file pone.0209068.s007.docx]

**S7 Table. Factor Level Legend for S2 Fig.**

| **Factor Level Name** | **Description** |
| --- | --- |
| DIAG_KI G1 | GROUP_1* |
| DIAG_KI G2 | GROUP_2* |
| DIAG_KI G3 | GROUP_3* |
| DIAG_KI G4 | GROUP_4* |
| DIAG_KI G5 | GROUP_5* |
| DIAG_KI G6 | GROUP_6* |
| DIAG_KI G7 | GROUP_7* |
| DIAG_KI G8 | GROUP_8* |
| DIAG_KI G9 | NOT_KNOWN |
| FUNC_STAT_TRR G1 | 10-20 PERCENT VERY SICK HOSPITALIZATION NECESSARY |
| FUNC_STAT_TRR G2 | 30-50 PERCENT REQUIRES CONSIDERABLE ASSISTANCE BUT DEATH NOT IMMINENT |
| FUNC_STAT_TRR G3 | 60-70 PERCENT PERFORMS ACTIVITIES OF DAILY LIVING WITH SOME ASSISTANCE |
| FUNC_STAT_TRR G4 | 80-100 PERCENT PERFORMS ACTIVITIES OF DAILY LIVING WITH NO ASSISTANCE |
| FUNC_STAT_TRR G5 | NOT APPLICABLE (PATIENT < 1 YEAR OLD) |
| FUNC_STAT_TRR G6 | NOT_KNOWN |
| FUNC_STAT_TRR G7 | PERFORMS ACTIVITIES OF DAILY LIVING WITH TOTAL ASSISTANCE. |
| MED_COND_TRR G1 | HOSPITALIZED NOT IN ICU |
| MED_COND_TRR G2 | IN INTENSIVE CARE UNIT |
| MED_COND_TRR G3 | NOT HOSPITALIZED |

*See S2 Table for variable values in this group.
