# Supplementary material for: Using machine learning and an ensemble of methods to predict kidney transplant survival
Source: PLoS One. 2019 Jan 9;14(1):e0209068. doi: 10.1371/journal.pone.0209068 (PMC6326487; doi:10.1371/journal.pone.0209068)
Supplement: S8 Table — The performance is calculated from 10 random samples of 80,000 training observations and 20,000 out-of-sample observations. (DOCX) [file pone.0209068.s008.docx]

**S8 Table. Concordance Index at Different Days after Transplant for the Proposed Model.**

| **Days** | **C-index** |
| --- | --- |
| 10 | 0.720 |
| 50 | 0.722 |
| 100 | 0.723 |
| 250 | 0.724 |
| 500 | 0.724 |
| 1000 | 0.724 |
| 1500 | 0.724 |
| 2000 | 0.724 |

The performance is calculated from 10 random samples of 80,000 training observations and 20,000 out-of-sample observations.
