# Supplementary material for: Using machine learning and an ensemble of methods to predict kidney transplant survival
Source: PLoS One. 2019 Jan 9;14(1):e0209068. doi: 10.1371/journal.pone.0209068 (PMC6326487; doi:10.1371/journal.pone.0209068)
Supplement: S9 Table — Performance from a random sample of 100,000 training observations and 25,000 out-of-sample observations. (DOCX) [file pone.0209068.s009.docx]

**S9 Table. Proposed Model Performance by Category.**

| **Group** | **5-Year Integrated Brier Score** | **C-index** |
| --- | --- | --- |
| AGE_DON: 0 - 24 | 0.051 | 0.730 |
| AGE_DON: 24 - 35 | 0.044 | 0.742 |
| AGE_DON: 35 - 44 | 0.057 | 0.717 |
| AGE_DON: 44 - 53 | 0.064 | 0.710 |
| AGE_DON: 53 - 84 | 0.090 | 0.688 |
| AGE: 0 - 36 | 0.023 | 0.670 |
| AGE: 36 - 47 | 0.038 | 0.702 |
| AGE: 47 - 55 | 0.061 | 0.652 |
| AGE: 55 - 62 | 0.079 | 0.640 |
| AGE: 62 - 90 | 0.109 | 0.637 |
| COLD_ISCH_KI: 0.01 - 1.4 | 0.037 | 0.732 |
| COLD_ISCH_KI: 1.4 - 8.4 | 0.053 | 0.731 |
| COLD_ISCH_KI: 8.4 - 15 | 0.070 | 0.707 |
| COLD_ISCH_KI: 15 - 22 | 0.073 | 0.703 |
| COLD_ISCH_KI: 22 - 99 | 0.077 | 0.703 |
| DIAB: NO | 0.046 | 0.727 |
| DIAB: YES | 0.095 | 0.650 |
| FUNC_STAT_TRR: 30-50 PERCENT REQUIRES CONSIDERABLE ASSISTANCE BUT DEATH NOT IMMINENT | 0.103 | 0.704 |
| FUNC_STAT_TRR: 60-70 PERCENT PERFORMS ACTIVITIES OF DAILY LIVING WITH SOME ASSISTANCE | 0.078 | 0.686 |
| FUNC_STAT_TRR: 80-100 PERCENT PERFORMS ACTIVITIES OF DAILY LIVING WITH NO ASSISTANCE | 0.055 | 0.730 |
| FUNC_STAT_TRR: NOT_KNOWN | 0.064 | 0.714 |
| HIST_DIABETES_DON: NO | 0.070 | 0.706 |
| HIST_DIABETES_DON: NOT_KNOWN | 0.039 | 0.732 |
| HIST_DIABETES_DON: YES | 0.108 | 0.682 |
| HIST_HYPERTENS_DON: NO | 0.055 | 0.728 |
| HIST_HYPERTENS_DON: NOT_KNOWN | 0.052 | 0.736 |
| HIST_HYPERTENS_DON: YES | 0.091 | 0.674 |
| PAYMENTSOURCE_AT_TRANSPLANT: MEDICAID | 0.051 | 0.727 |
| PAYMENTSOURCE_AT_TRANSPLANT: MEDICARE | 0.072 | 0.699 |
| PAYMENTSOURCE_AT_TRANSPLANT: SOME PRIVATE BY PRIMARY OR SECONDARY | 0.052 | 0.741 |

Performance from a random sample of 100,000 training observations and 25,000 out-of-sample observations.
