# Supplementary material for: Using machine learning and an ensemble of methods to predict kidney transplant survival
Source: PLoS One. 2019 Jan 9;14(1):e0209068. doi: 10.1371/journal.pone.0209068 (PMC6326487; doi:10.1371/journal.pone.0209068)
Supplement: S10 Table — 10 random samples of 80,000 training observations and 20,000 out-of-sample observations. **Donor variables that were removed: AGE_DON, COD_CAD_DON, COLD_ISCH_KI, DEATH_MECH_DON, HIST_DIABETES_DON, and HIST_HYPERTENS_DON. (DOCX) [file pone.0209068.s010.docx]

**S10 Table. Additional Model Testing.**

| **Model** | **5-Year C-index** | **5-Year Integrated Brier Score** |
| --- | --- | --- |
| EPTS for Adult Recipients and Deceased Donors Using the Same Cross-Validation Data as the Proposed Model | 0.665 | Not Calculated |
| Proposed Model Using PMM Imputation for Adult Recipients and Deceased Donors and without Donor Variables** | 0.689 | 0.078 |

10 random samples of 80,000 training observations and 20,000 out-of-sample observations. **Donor variables that were removed: AGE_DON, COD_CAD_DON, COLD_ISCH_KI, DEATH_MECH_DON, HIST_DIABETES_DON, and HIST_HYPERTENS_DON.
