# Supplementary material for: Using machine learning and an ensemble of methods to predict kidney transplant survival
Source: PLoS One. 2019 Jan 9;14(1):e0209068. doi: 10.1371/journal.pone.0209068 (PMC6326487; doi:10.1371/journal.pone.0209068)
Supplement: S11 Table — C-index based on 10 random samples of 80,000 training observations and 20,000 out-of-sample observations. Using a paired two sample Student's t-test, we reject the null hypothesis that the difference in model performance means is equal to 0 (p-value = 2.4x10^-11^). We also used a Shapiro-Wilk normality test and an F test for equality of variances to verify the assumptions of the t-test (Shapiro-Wilk p-value of 0.654 and 0.298 for the proposed model data and EPTS model data respectively; hence we don’t reject the null hypothesis of normally distributed model performance results. F test p-value of 0.605; hence we don’t reject the null hypothesis of equality of model performance variance). (DOCX) [file pone.0209068.s011.docx]

**S11 Table. Proposed Model and EPTS Model Cross-Validation Results in Table 2.**

| **Test** | **Proposed Model** | **EPTS Model** |
| --- | --- | --- |
| 1 | 0.721 | 0.698 |
| 2 | 0.727 | 0.701 |
| 3 | 0.724 | 0.694 |
| 4 | 0.724 | 0.695 |
| 5 | 0.721 | 0.692 |
| 6 | 0.715 | 0.685 |
| 7 | 0.727 | 0.700 |
| 8 | 0.720 | 0.695 |
| 9 | 0.729 | 0.702 |
| 10 | 0.730 | 0.702 |

C-index based on 10 random samples of 80,000 training observations and 20,000 out-of-sample observations. Using a paired two sample Student's t-test, we reject the null hypothesis that the difference in model performance means is equal to 0 (p-value = 2.4x10^-11^). We also used a Shapiro-Wilk normality test and an F test for equality of variances to verify the assumptions of the t-test (Shapiro-Wilk p-value of 0.654 and 0.298 for the proposed model data and EPTS model data respectively; hence we don’t reject the null hypothesis of normally distributed model performance results. F test p-value of 0.605; hence we don’t reject the null hypothesis of equality of model performance variance).
