# Supplementary material for: Using machine learning and an ensemble of methods to predict kidney transplant survival
Source: PLoS One. 2019 Jan 9;14(1):e0209068. doi: 10.1371/journal.pone.0209068 (PMC6326487; doi:10.1371/journal.pone.0209068)
Supplement: S1 Text — (DOCX) [file pone.0209068.s012.docx]

**S1 Text. Grouping Values for the Variable Kidney Diagnosis**

To group and reduce the number of possible values for the variable kidney diagnosis:

1. Use domain knowledge to initially assign related values into the same group.
2. Create binary variables (dummy variables) corresponding to each of the remaining values for the categorical variable.
3. Build a Cox proportional hazards model to predict recipient post-transplant survival using the binary variables from step 2, in addition to other relevant variables to control for, such as recipient age.
4. Sort the coefficients for the binary variables corresponding to the categorical variable from the Cox model into a prespecified number of quantiles, representing the new categorical variable values.
