# Supplementary material for: Using machine learning and an ensemble of methods to predict kidney transplant survival
Source: PLoS One. 2019 Jan 9;14(1):e0209068. doi: 10.1371/journal.pone.0209068 (PMC6326487; doi:10.1371/journal.pone.0209068)
Supplement: S2 Text — The analysis was undertaken using the statistical software R version 3.3.2 as well as several key packages listed in the references [24–30]. (DOCX) [file pone.0209068.s013.docx]

**S2 Text. Software.** The analysis was undertaken using the statistical software R version 3.3.2 as well as several key packages listed in the references [24-30]. The random survival forests predictive model was implemented using the function, cforest, of the R package “party” and the Cox predictive model was implemented using the function, coxph, in the R package “survival”. The following parameters were used in the cforest function: mtry=4, ntree = 800, teststat = ‘quad’, testtype = ‘Univ’, mincriterion = .3, replace = FALSE, fraction =0.632.
