# Supplementary figures and images for: Using machine learning and an ensemble of methods to predict kidney transplant survival
Source: PLoS One. 2019 Jan 9;14(1):e0209068. doi: 10.1371/journal.pone.0209068 (PMC6326487; doi:10.1371/journal.pone.0209068)

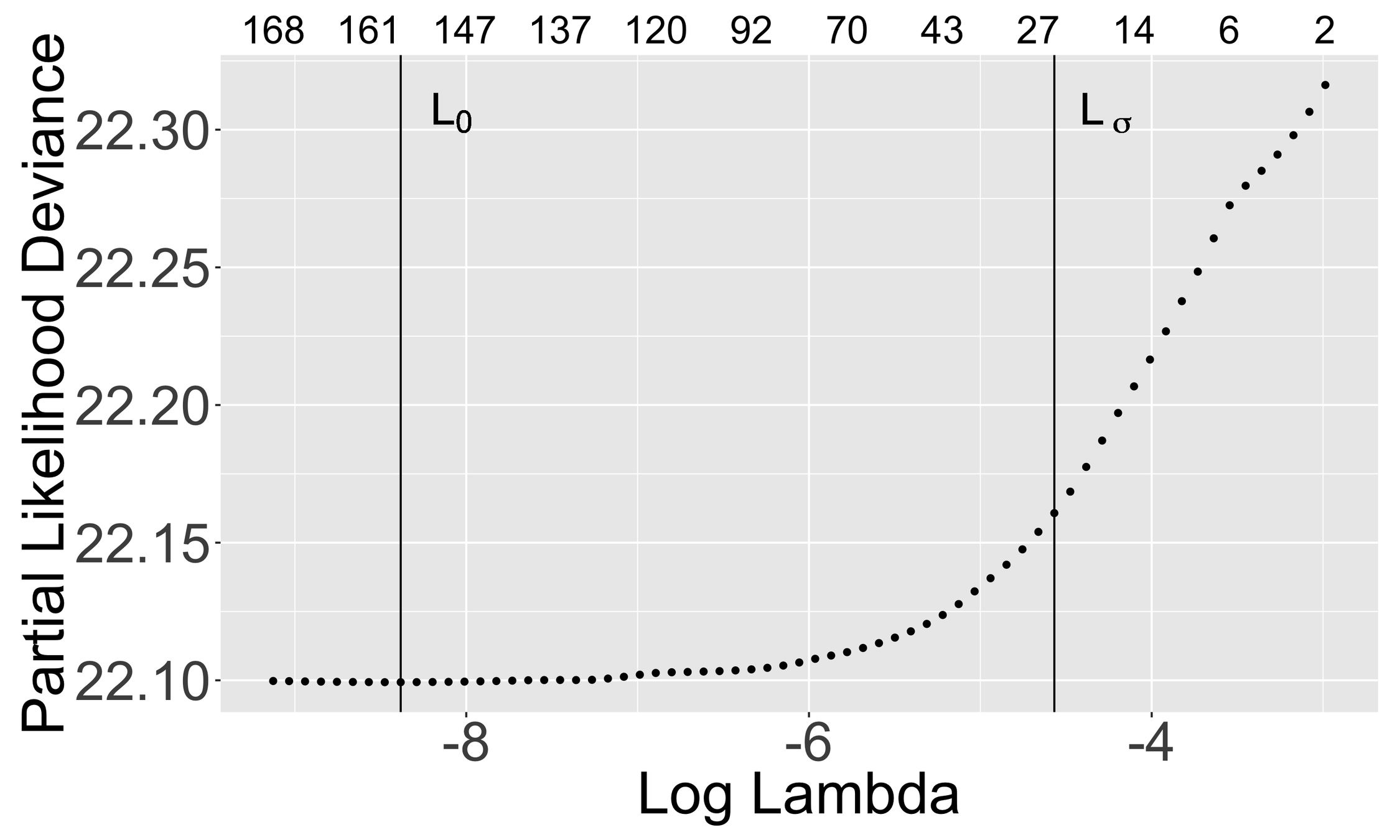

Supplement: S1 Fig — (TIF) [file pone.0209068.s014.tif]

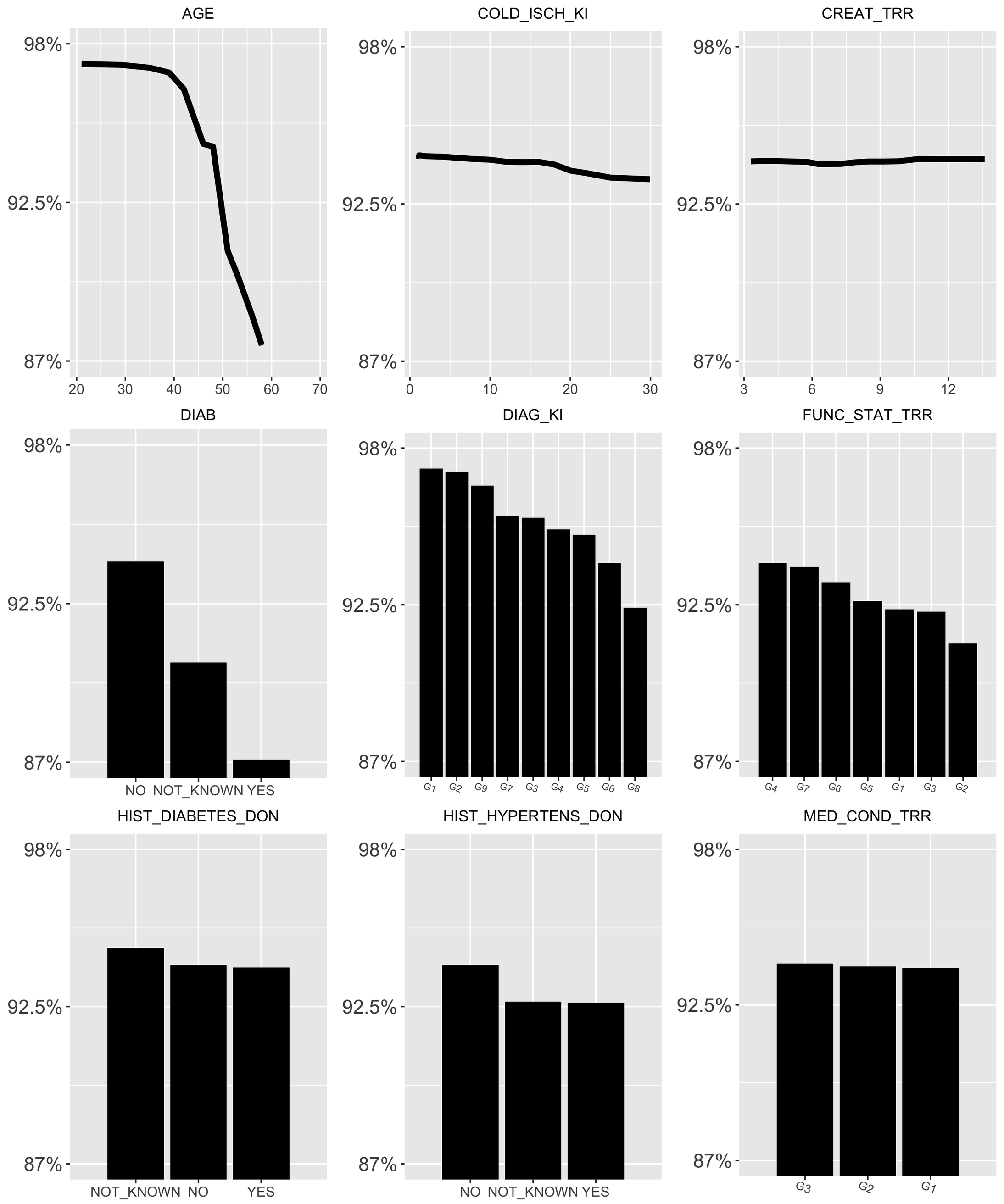

Supplement: S2 Fig — In each plot, variable values not shown are held constant and listed in S6 Table. The model was trained on 100,000 random training observations. (TIF) [file pone.0209068.s015.tif]
